# Plate Versus Intramedullary Fixation Care of Displaced Midshaft Clavicular Fractures: A Meta-Analysis of Prospective Randomized Controlled Trials

**DOI:** 10.1097/MD.0000000000001792

**Published:** 2015-10-16

**Authors:** Xin-Hua Wang, Lin Cheng, Wei-Jun Guo, A-Bing Li, Guang-Jun Cheng, Tao Lei, You-Ming Zhao

**Affiliations:** From the Department of Orthopaedics Surgery, The Second Affiliated Hospital of Wenzhou Medical University, Wenzhou, Zhejiang (X-HW, W-JG, A-BL, G-JC, TL, Y-MZ); and Department of Rheumatology and Immunology, The Second Affiliated Hospital of Anhui Medical University, Hefei, Anhui, People's Republic of China (LC).

## Abstract

In recent decades, there has been a growing trend to the operative treatment of displaced midshaft clavicular fractures. Open reduction and internal plate fixation, and intramedullary nailing fixation are 2 of the widely used techniques for operative treatment, but the optimal fixation method for these types of fractures remains a topic of debate. The objective of this study was to determine the effectiveness of plate fixation versus intramedullary nailing fixation for displaced midshaft clavicle fractures by comparing their clinical results.

Literature searches of the Pubmed, EMBASE, and Web of Science were performed from 1966 to April, 2015. Only randomized controlled clinical trials comparing plate and intramedullary nailing treatment for displaced midshaft clavicle fractures were included. Literature was screened, data were extracted, and methodological quality of the eligible trials was assessed by 2 independent reviewers accordingly.

Seven randomized controlled trials involving 421 patients were included. Compared to intramedullary nailing fixation, plate fixation had a relatively longer mean surgical time and a trend towards a faster functional improvement during the first 6 months after surgery; apart from this, the pooled results revealed no significant differences in functional scores after 6 months postoperatively, complication rate and patients’ satisfaction between plate fixation and intramedullary fixation.

Our results demonstrated that these 2 methods were comparable and safe in the treatment of displaced midshaft clavicle fractures. We advocate both techniques for the treatment of displaced midshaft clavicle fractures, and the superior surgical technique was those that the surgeon was originally trained to perform.

## INTRODUCTION

Clavicle fractures, accounting for about 2.6% of total body fractures and 34% to 45% of shoulder girdle injuries in adults, are one of the commonest bone injuries in the body.^1–3^ About 69% to 81% of clavicle fractures are in the middle one-third of the clavicle, which is the thinnest part and entails the least soft tissue, 17% in the lateral one-third, and 2% in the medial one-third.^4–6^ Recently, there has been a growing trend to the operative treatment of displaced midshaft clavicular fractures. Open reduction and internal plate fixation, and intramedullary nailing fixation are 2 of the widely used techniques for operative treatment,^7–11^ but the optimal fixation method for these types of fractures remains a topic of debate.

Previous meta-analyses have compared plate fixation versus intramedullary nailing fixation for the treatment of midshaft clavicle fractures.^12,13^ However, the relatively small sample size in each published study made the results inconclusive. Moreover, several relevant studies on this topic have been published in recent years, which make the present meta-analysis more precise.

The objective of this study was to determine the effectiveness of plate fixation versus intramedullary nailing fixation for displaced midshaft clavicle fractures by comparing their clinical results reported in all the related prospective randomized controlled trials. The primary outcomes were the Disabilities of the Arm, Shoulder, and Hand (DASH) score and the Constant–Murley (CM) score^14–16^; the secondary outcomes included postoperative complications, duration of surgery, and patient satisfaction.

## METHODS AND MATERIALS

This study was performed with guidance from the Cochrane Handbook for Systematic Reviews of Interventions and the Preferred Reporting Items for Systematic Reviews and Meta-Analyses statement.^17,18^ Because the present meta-analysis was performed based on previous published studies, ethical approval and patient consent were not necessary.

### Inclusion and Exclusion Criteria

The search results were screened based on the following inclusion criteria: the studies had to be randomized or quasi-randomized controlled clinical trials design on patients with displaced midshaft clavicular fractures that had occurred less than 2 weeks; the studies had to compare plate fixation with intramedullary nail fixation; the patients had to be aged at least 16 years; and comparison of functional outcome, measured with the DASH score and the CM score, postoperative complications. The exclusion criteria included the following: a pathologic fracture or having pre-existing shoulder abnormalities; studies concerning adolescent fractures; nonrandomized studies, review literature, repeated reports, retrospective studies, or case reports; and did not report outcomes of interest.

### Search Strategy and Study Selection

The search strategies are shown in Table 1. Electronic literature databases used for searching included Pubmed, EMBASE, and Web of Science (up to April, 2015). The search was performed without language restrictions, but was limited to humans. The function of “related article” was also used for the search. In addition, the reference lists of identified studies, and previous systematic reviews and meta-analyses were manually checked to include other potentially eligible trials. This process was performed iteratively until no additional articles could be identified by 2 authors (X-HW and LC) independently; any disagreement was discussed and resolved with the third independent author (W-JG).

**TABLE 1:**
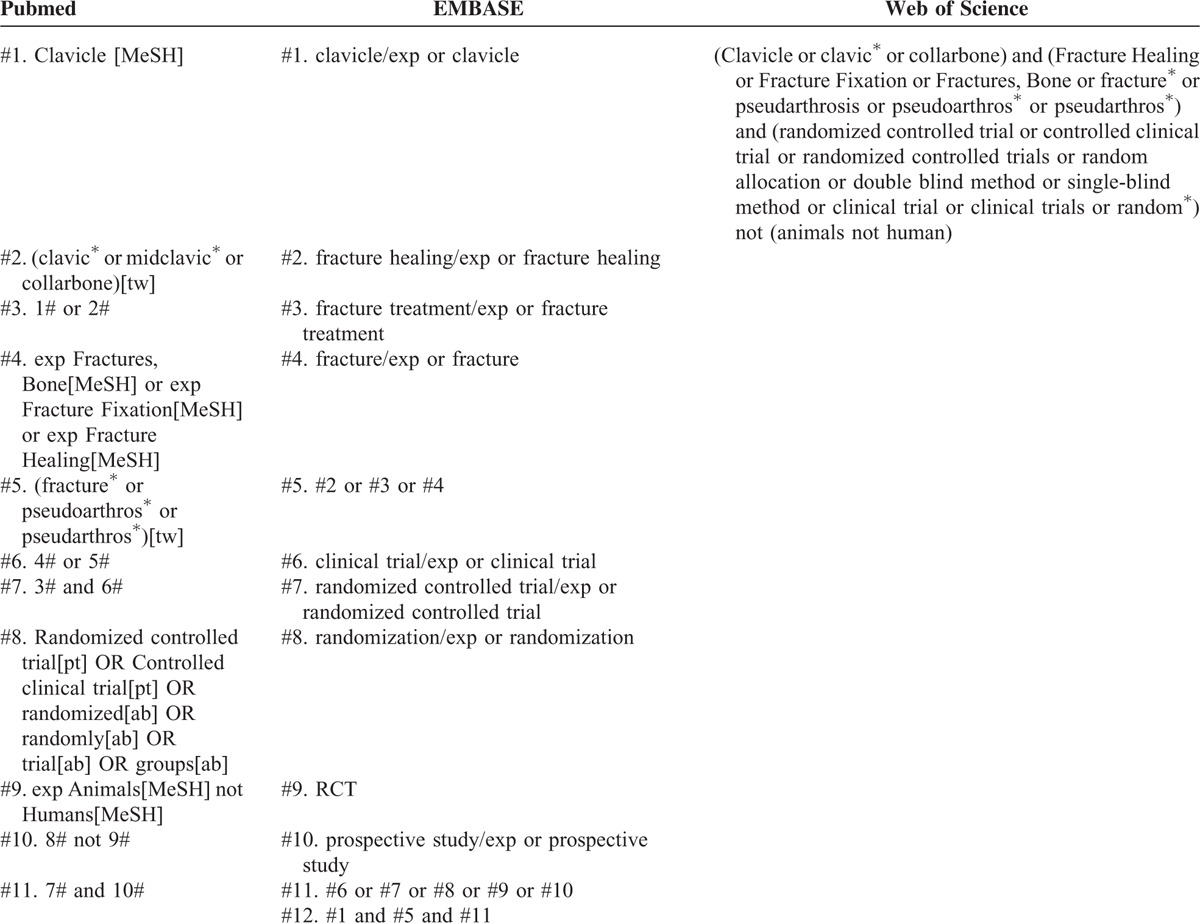
Search Strategy

### Data Extraction

Two authors (X-HW and A-BL) independently extracted data for analysis, and the third author checked the consistency between them. A standard data extracted form was used, including the first author's last name, publication year, country where the study was performed, follow-up duration, sample size, characteristics of patients, interventions, functional outcomes, postoperative complications, duration of surgery, and patient satisfaction. If necessary, the primary authors were contacted to retrieve additional information.

### Risk of Bias Assessment

The methodological quality of the studies was evaluated independently by 2 authors (X-HW and LC); the reviewers assessed the risk of bias of the included RCTs according to the Cochrane Handbook for Systematic Reviews of Interventions: random sequence generation; allocation concealment; blinding of participants and personnel; blinding of outcome assessment; incomplete outcome data addressed; selective reporting; and other bias. Additionally, judgments of the reviewers were classified as “low risk,” “high risk,” or “unclear risk” of bias.

### Statistical Analysis

Estimates of treatment effect were expressed as relative risk (RR) for dichotomous outcomes and standard mean difference (SMD) for continuous outcomes, both with 95% confidence intervals (CIs). For studies that did not present standard deviations, the standard deviations were calculated from the *P* value or CI following the guidance of the Cochrane Handbook for Systematic Reviews of Interventions.^17^ Chi-square and *I*^2^ statistics were used to evaluate the statistical heterogeneity; *P* <0.10 for the chi-square test or for *I*^2^ >50% was considered as significantly statistical heterogeneity.^19^ A fixed-effects model was used when the heterogeneity was not significant, and a random-effects model was adopted if statistically significant heterogeneity was present. Sensitivity analysis was performed by removing 1 study each time to explore potential sources of heterogeneity and to test the stability of pooled results. Statistical analyses were conducted using RevMan 5.3.5 software (The Nordic Cochrane Center, Denmark). All reported *P* values were 2-sided, and *P* <0.05 was determined as statistically significant.

## RESULTS

### Included Studies and Risk of Bias Assessment

**FIGURE 1:**
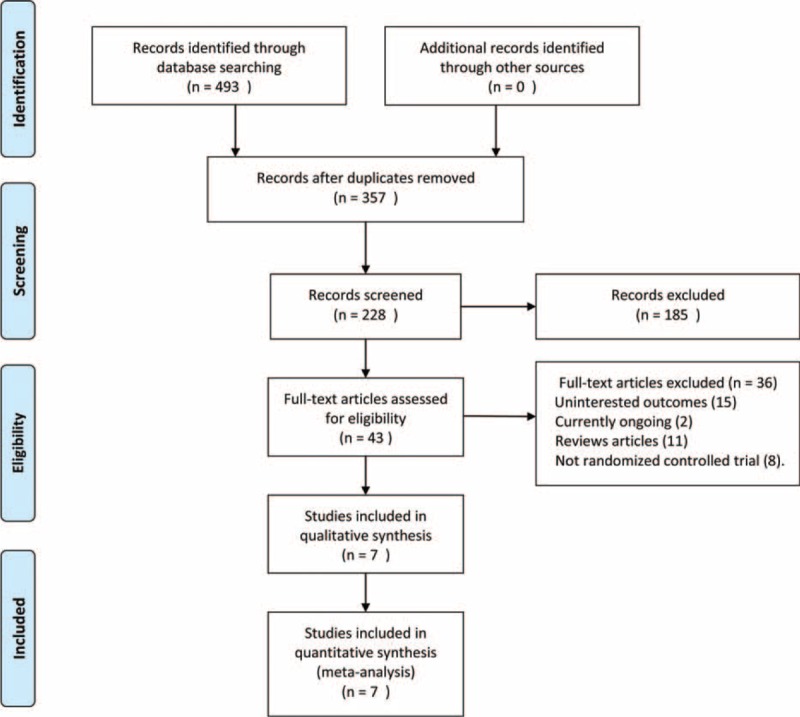
The selection of literatures for included studies.

A total of 493 potential records were identified from the databases, and 185 studies were excluded after screening the title and the abstract. In all, 43 full-text articles were assessed for eligibility, of which 8 were excluded for nonrandomized clinical trials and 2 for currently ongoing studies; 15 trials were excluded due to the uninteresting outcomes and 11 studies were excluded because of review articles. The remaining 7 studies^20–26^ were included in this meta-analysis. Of the 7 studies, 1 study^24^ was a multicenter randomized controlled clinical trial and 6 were from a single investigational site; all randomized clinical trials enrolled patients with completely displaced midshaft clavicular fractures (Figure 1).

A total of 421 patients were included, 208 of whom were treated with plate fixation and the others with intramedullary nailing fixation. The studies were performed in various countries with no significant differences in baseline demographics between the intramedullary nailing and plate groups, and individuals enrolled in all 6 studies were basically homogeneous; all the participants were followed up for at least 12 months (Table 2).

**TABLE 2:**
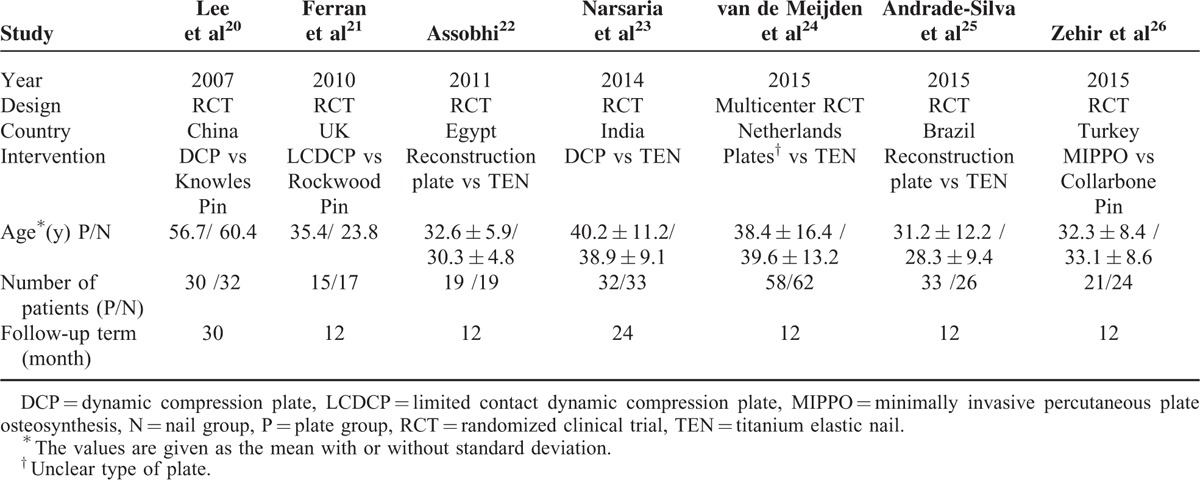
Characteristics of the Included Studies

**FIGURE 2:**
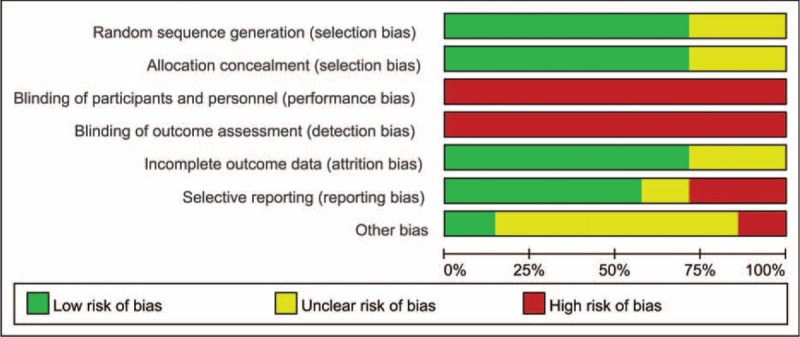
Risk of bias graph: review authors’ judgments about each risk of bias item presented as percentages across all included studies.

**FIGURE 3:**
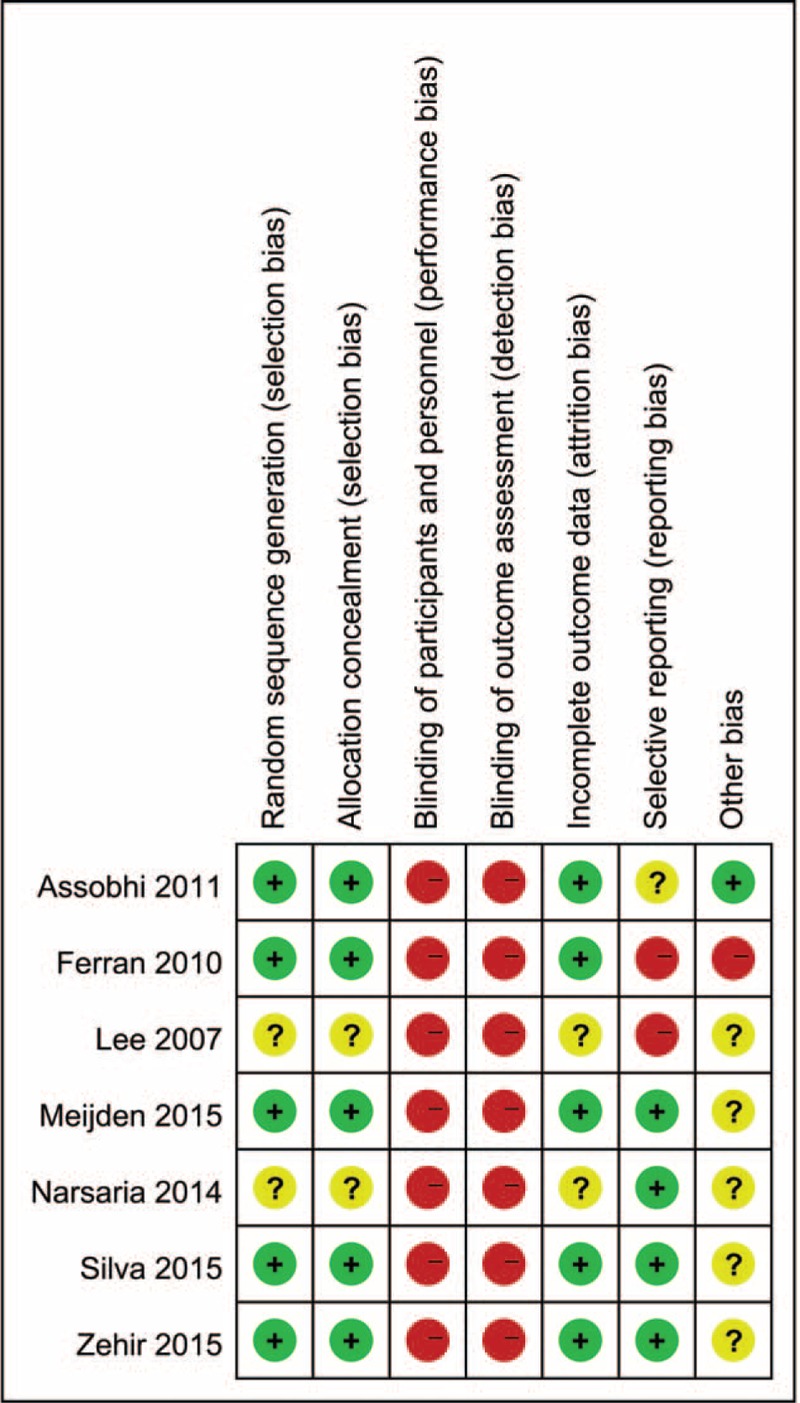
Risk of bias summary: review authors’ judgments about each risk of bias item for each included study.

The risk of bias was demonstrated graphically in Figure 2 and summarized in Figure 3. The randomization technique was mentioned in 5 studies,^21,22,24–26^ and information of allocation concealment was not available for 2 studies.^20,23^ Blinding was hardly used in open surgery trials and no 1 study was blinded in the assessment of outcome; thus, the term “blinding of outcome assessment” was assessed as “high risk” for all the 7 studies.

### Functional Outcomes

Three studies^24–26^ provided both mean value and standard deviation of the DASH scores at 6 months and last follow-up postoperatively. Three studies reported the mean value and standard deviation of CM scores at 3 weeks^22–24^ and 6 months^22,24,25^ postoperatively, and 2 studies^22,24^ provided CM scores at 3 months; meanwhile, the actual numbers of mean value and standard deviation for CM scores at the last follow-up postoperatively were found in 5 studies.^21–25^

Due to the statistically undetectable heterogeneity, meta-analysis for the functional outcomes during the first 6 months after surgery was cancelled and a descriptive review was conducted instead. The study by Assobhi^22^ showed significant higher CM scores at the 6th week in the intramedullary group than in the plate group. Conversely, Narsaria et al^23^ reported that the plate group offers significantly higher CM scores at the 2nd month. Van der Meijden et al^24^ suggested that plate fixation resulted in more rapid improvement in the DASH score and led to better subjective function during the first 6 months after surgery.

**FIGURE 4:**
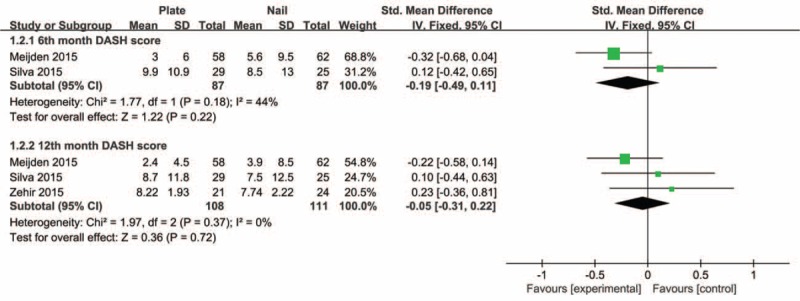
Forest plot of DASH scores at 6 months and last follow-up postoperatively. DASH = Disabilities of the Arm, Shoulder, and Hand.

**FIGURE 5:**
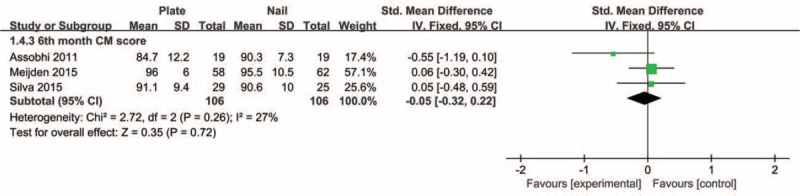
Forest plot of Constant–Murley scores at 6 months postoperatively.

**FIGURE 6:**
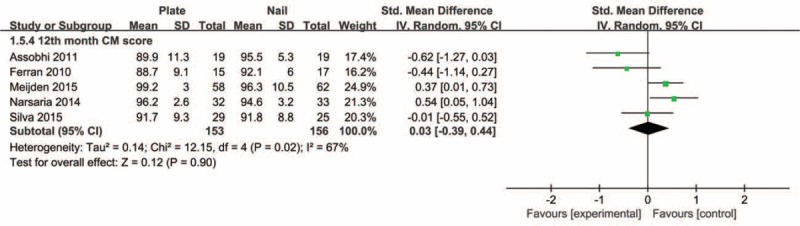
Forest plot of Constant–Murley scores at final follow-up postoperatively.

Pooled data of DASH scores at 6 months and last follow-up postoperatively showed that the plate group was not significantly different in comparison with the intramedullary nail group (SMD −0.19, 95% CI −0.49 to 0.11, *P* = 0.22; SMD −0.05, 95% CI −0.31 to 0.22, *P* = 0.72, respectively) (Figure 4). There was no significant heterogeneity detected among these studies. Also, the aggregated results suggested that there were no significant differences between groups for the CM scores at 6 months or last follow-up postoperatively (SMD −0.05, 95% CI −0.32 to 0.22, *P* = 0.72; SMD 0.03, 95% CI −0.39 to 0.44, *P* = 0.90, respectively). Moderate heterogeneity was detected among these studies (*I*^2^ = 67%, *P* = 0.02). Subsequently, we performed sensitivity analysis to explore potential sources of heterogeneity. Exclusion of the trial conducted by Assobhi^22^ reduced the heterogeneity, but did not materially alter the pooled results (Figures 5 and 6).

### Postoperative Complications

With the inconsistent definition of the complications across all included studies, the meta-analysis of overall complications was inappropriate. Thus, only the major adverse events including incidence of fixation failure, infection, nonunion, symptomatic hardware, hypertrophic scar, and refracture after implant removal were incorporated into the meta-analysis to summarize the evaluation. Six studies^20–23,25,26^ reported the incidence of fixation failure, with a low frequency in both groups.

**FIGURE 7:**
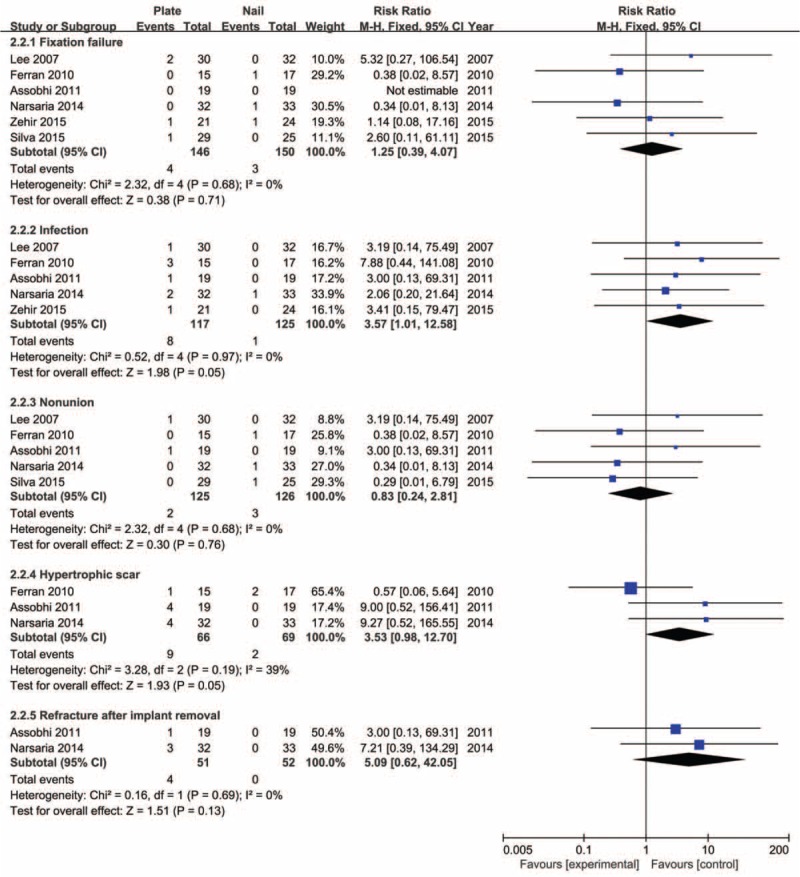
Forest plot for incidence of fixation failure, infection, nonunion, hypertrophic scar, and refracture after implant removal.

The meta-analysis for pooled results showed no significant discrepancy (RR 1.25, 95% CI 0.39–4.07, *P* = 0.71) without any significant heterogeneity (*I*^2^ = 0%, *P* = 0.68). Five studies^20–23,26^ dealt with the outcome measure of infection, and all events were superficial infections. The heterogeneity test indicated low variance across studies (*I*^2^ = 0%, *P* = 0.97). And then, a fixed-effects model was adopted; meta-analysis showed no significant differences between groups (RR 3.57, 95% CI 1.01–12.58, *P* = 0.05). All 6 studies reported the incidence of nonunion, although it was an uncommon occurrence, with an incidence rate of less than 3%. Only 5 studies^20–23,25^ could provide actual data, and the pooled results showed no significant differences between both groups (RR 0.83, 95% CI 0.24–2.81, *P* = 0.76) without any heterogeneity (*I*^2^ = 0%, *P* = 0.68). Three studies^21–23^ provided outcomes of hypertrophic scar and 2 studies^22,23^ provided outcomes of refracture after implant removal. Both results of hypertrophic scar and refracture after implant removal showed no significant difference between the 2 groups without any heterogeneity (RR 3.53, 95% CI 0.98–12.70, *P* = 0.05; RR 5.09, 95% CI 0.62–42.05, *P* = 0.13, respectively) (Figure 7).

**FIGURE 8:**
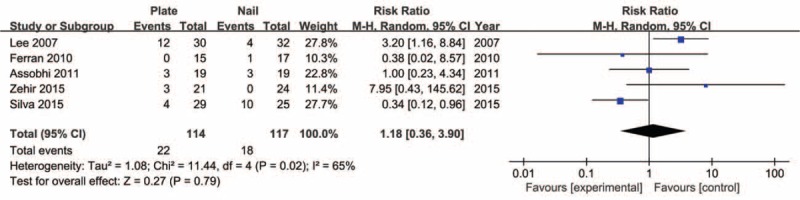
Forest plot for incidence of symptomatic hardware.

Five studies^20–22,25,26^ reported the incidence of postoperative symptomatic hardware. The meta-analysis investigated no significant differences (RR 1.18, 95% CI 0.36–3.90, *P* = 0.79) with moderate heterogeneity (*I*^2^ = 65%, *P* = 0.02). Sensitivity analysis by exclusion of the trial conducted by Lee et al^20^ resolved the heterogeneity without materially altering the pooled results (Figure 8).

### Duration of Surgery and Patient Satisfaction

No attempt at meta-analysis for duration of surgery and patient satisfaction was made due to the incompatible data forms, and a descriptive review was performed. The mean operative time was significantly longer in the plate group compared with the nail group in 5 studies.^20,22–25^ One study^24^ reported there was no difference between the groups in terms of satisfaction with the cosmetic appearance; another research^25^ described both groups obtained satisfactory therapeutic results without significant differences.

## DISCUSSION

Both open reduction, and internal plate fixation and intramedullary fixation are the standard surgical techniques for treating displaced midshaft clavicular fractures. Apart from these, there are multiple choices about plating and intramedullary devices. Reconstruction plate^25,27^ or precontoured plate, including dynamic compression plate^23,28^ and locked compression plate,^29,30^ are widely applied in the plating fixation, whereas Knowles pinning,^20,31^ elastic stable intramedullary nailing,^24^ and Rockwood pin^21^ are commonly used for intramedullary fixation. In the present study, we could not perform a subgroup analysis concerning different forms of plate and intramedullary devices, which were restricted by the insufficient samples. With advanced implants, prophylactic antibiotics, and better soft-tissue handling, plate fixation and intramedullary fixation have been reliable techniques, and previous literature has shown that both methods produce excellent results in midshaft clavicular fractures.^24,25,32^ Despite proposed benefits, plating and intramedullary nailing methods both have their own advantages and disadvantages. Biomechanically, plate fixation is superior to intramedullary fixation,^33^ and plate fixation can be allowed full range of motion by providing rigid fixation, which is favorable for early rehabilitation protocols. Additionally, plate fixation is technically less exacting.^33^ Disadvantages of plate fixation include the necessity for increased exposure and soft-tissue stripping, increased risk of damage to the supraclavicular nerve, slightly higher infection rates, hypertrophic scars, and the risk of refracture after plate removal.^32,34,35^

Compared with plate fixation, intramedullary technique has potential advantages such as less invasive, shorter hospital stay, elastic stability, less blood loss, and more cosmetic satisfaction.^22,23,36^ Its main disadvantages include skin irritation, implant migration, and frequent need for implant removal. Nevertheless, the question of which form of fixation is superior remains, especially with a myriad of options available for both methods.

High-quality evidence from 7 randomized studies showed similar curative effect after plate fixation and intramedullary fixation. No significant difference in the primary functional outcomes was noted between the 2 surgical interventions. The pooled DASH and CM scores at any period postoperatively were parallel. As the duration of follow-up varied in the included studies, special note should be made that the present study contained a subgroup analysis of the functional outcomes at the time of final follow-up. However, previous study results indicated that patients reached a steady state of shoulder function 1 year after surgery.^11^ Therefore, the overall results were reliable.

There is a big argument about which surgical treatment is associated with a faster functional recovery. The results vary widely from study to study. In the present study, the result seems to give more support to the plate fixation. Unfortunately, without sufficient original data, we cannot perform subgroup analysis of functional outcomes in the early stage after operation; further studies on this topic are warranted.

Our meta-analysis revealed that the frequency of postoperative complications was similar between the interventions. The concept of “symptomatic hardware” was defined as prominent implant irritation or protrusions. The commonest complications including symptomatic hardware, fixation failure, superficial infection, and hypertrophic scar and refracture, and most of the complications were hardware-related. In general, the incidence of complications was lower. However, Lee et al^20^ reported a high incidence of symptomatic hardware with 12 of 30 older patients suffering prominent plates and screws problems, probably owing to the poor skin and bone quality of older patients. Two other studies^24,25^ reported a high rate of implant-related pain and protrusion of the titanium elastic nails, which have been demonstrated in previous studies.^36,37^ Lots of solutions to this problem can be recommended, such as cutting the nail close to the bone cortex,^38^ the use of an end cap, retrograde nailing technique, and less prominent implants.^22,36,39^

The risk of refracture following implant removal was identified in previous studies^34,35^; our results showed a higher rate refracture in the plate group than in the nail group without statistically significant differences. Moreover, only 2 studies reported outcomes of refracture with a small sample size, and we were not able to draw any specific conclusions. But steps could be easily taken to prevent refracture after implant removal, in agreement with other researchers. It was also necessary to caution patients to avoid high-risk activities during the first months following removal.^24^

We identified several published reviews on this topic. The results and conclusions of those published reviews varied, which was partly in accordance with ours. The current study adds 3 new RCTs that were not previously available; what is more, we applied more rigorous methodology, restricting the included studies to randomized trials, and performed a more comprehensive literature search. Therefore, the conclusion gained in this study was relatively more convincing.

There are some limitations of this study. Firstly, technically, although the recruited studies were all randomized controlled trials, the lack of inadequate allocation concealment and failure to blind the outcome assessor in the majority of trials, which could lead to over-reporting of the treatment effect and selection or allocation biases, likely affected the study results. Secondly, the fracture pattern was found to be significantly related to implant failure^40^; similar to a previous study,^12^ our meta-analysis also failed to show fracture type-specific effects between the 2 surgical techniques because of the limited data of the studies. Finally, only 7 studies with 421 participants were included in the review, which may weaken the strength of evidence and clinical significance of this analysis. Moreover, despite our best efforts in using multiple search methods, we were not able to detect all eligible existing trials with results that were applicable to our meta-analysis. Therefore, the conclusions should be interpreted with caution. Further research entailing high-quality randomized controlled, multicenter trials with fracture type-specific design is required to address the key clinical questions regarding the optimum fixation treatment in the management of displaced midshaft clavicular fractures in adults.

## CONCLUSIONS

Intramedullary nailing showed its advantage over plate in mean surgical time, whereas plate fixation tends to provide more rapid functional improvement during the first 6 months after surgery. However, there was no significant difference of functional outcomes, complications and patient satisfaction between the 2 groups after 6 months postoperatively. We concluded that, on the basis of 7 high-quality evidences, these 2 methods were comparable and safe in the treatment of displaced midshaft clavicle fractures. We advocate both techniques for the treatment of displaced midshaft clavicle fractures and the superior surgical technique was those that the surgeon was originally trained to perform.
